# Development of Gene Expression-Based Random Forest Model for Predicting Neoadjuvant Chemotherapy Response in Triple-Negative Breast Cancer

**DOI:** 10.3390/cancers14040881

**Published:** 2022-02-10

**Authors:** Seongyong Park, Gwansu Yi

**Affiliations:** Korean Advanced Institute of Science and Technology, Daejeon 34141, Korea; sypark0215@kaist.ac.kr

**Keywords:** neoadjuvant chemotherapy (NAC), triple negative breast cancer (TNBC), machine learning (ML), random forest (RF), predictive biomarker, pathological complete response (pCR), residual disease (RD)

## Abstract

**Simple Summary:**

Only 20–50% of patients with triple negative breast cancer achieve a pathological complete response from neoadjuvant chemotherapy, a strong indicator of patient survival. Therefore, there is an urgent need for a reliable predictive model of the patient’s pathological complete response prior to actual treatment. The purpose of this study was to develop such a model based on random forest recursive feature elimination and to benchmark the performance of the proposed model against existing predictive models. Our study suggests that an 86-gene-based random forest model associated to DNA repair and cell cycle mechanisms can provide reliable predictions of neoadjuvant chemotherapy response in patients with triple negative breast cancer.

**Abstract:**

Neoadjuvant chemotherapy (NAC) response is an important indicator of patient survival in triple negative breast cancer (TNBC), but predicting chemosensitivity remains a challenge in clinical practice. We developed an 86-gene-based random forest (RF) classifier capable of predicting neoadjuvant chemotherapy response (pathological Complete Response (pCR) or Residual Disease (RD)) in TNBC patients. The performance of pCR classification of the proposed model was evaluated by Receiver Operating Characteristic (ROC) curve and Precision Recall (PR) curve. The AUROC and AUPRC of the proposed model on the test set were 0.891 and 0.829, respectively. At a predefined specificity (>90%), the proposed model shows a superior sensitivity compared to the best performing reported NAC response prediction model (69.2% vs. 36.9%). Moreover, the predicted pCR status by the model well explains the distance recurrence free survival (DRFS) of TNBC patients. In addition, the pCR probabilities of the proposed model using the expression profiles of the CCLE TNBC cell lines show a high Spearman rank correlation with cyclophosphamide sensitivity in the TNBC cell lines (SRCC =0.697, *p*-value =0.031). Associations between the 86 genes and DNA repair/cell cycle mechanisms were provided through function enrichment analysis. Our study suggests that the random forest-based prediction model provides a reliable prediction of the clinical response to neoadjuvant chemotherapy and may explain chemosensitivity in TNBC.

## 1. Introduction

Triple negative breast cancer (TNBC) is a particularly difficult form of breast cancer to treat because of its rapid growth and high recurrence rate [1]. It accounts for about 15% of all invasive breast cancers and usually has a high histological grade with a low long term survival rate [2]. Compared to the successful application of targeted therapies for other types of breast cancer, targeted therapy for TNBC is difficult due to the lack of major receptors in breast cancer such as ER/PR/HER2 receptors [3,4]. Several targeted therapies for TNBC patients have recently been introduced [5,6,7], but due to the limited applicability of these therapies [8], cytotoxic chemotherapy remains the mainstay of treatment [9].

Due to the aggressive tumor growth in TNBC, NeoAdjuvant Chemotherapy (NAC), which refers to preoperative chemotherapy, is often considered as a first-line treatment for TNBC patients. The main treatment goal of NAC is to achieve a pathological complete response (pCR) and pCR is defined as the complete disappearance of all invasive carcinoma cells in the breast and axillary lymph nodes, which is assessed pathologically in the resected tissue after neoadjuvant chemotherapy [10]. Patients who do not achieve pCR are refer to as having Residual Disease (RD).

The pCR by NAC has been demonstrated to be a strong prognostic factor for TNBC patients [11,12,13]. Therefore, pCR by NAC generally is considered as an appropriate surrogate endpoint for TNBC patients [12] and clinical studies have shown improved survival in TNBC patients who achieved pCR by NAC [14]. Unfortunately, not all patients receiving NAC could achieve pCR and in fact, only about 20–50% of patients with TNBC achieve pCR by NAC [11,12,14,15]. In other words, some patients are experiencing unnecessary side effects that can lead to loss of alternative treatment due to an inadequate and ineffective treatment. Therefore, there is an urgent need for a prediction model of the NAC response in TNBC.

Several previous studies have proposed gene expression-based predictive models to address this issue [16,17,18,19,20,21]. For example, Liedtke et al. reported that a high GGI (Genomic Grade Index) was found to be correlated with increased sensitivity to neoadjuvant chemotherapy [22]. Hess et al. [23], proposed DLDA30 to predict the pCR of patients who received preoperative weekly paclitaxel and fluorouracil-doxorubicin-cyclophosphamide (T/FAC) chemotherapy based on Diagonal Linear Discriminant Analysis (DLDA). Hatzis et al. [18] proposed a prediction model for the NAC response by Taxane + Anthracycline based on the Threshold Gradient Directed Regularization (TGDR) method [24,25]. Masanori Oshi et al. [19], proposed a three-gene-based predictive biomarker for pCR prediction after NAC in TNBC. The same authors also proposed a five-gene-based predictive biomarker for pCR prediction after NAC in ER+/HER2- breast cancer [20]. Fu et al. [21] proposed an immune-associated genomic signature to predict pCR after Neoadjuvant paclitaxel and anthracycline based chemotherapy in breast cancer. Fournier et al. [26] proposed a two-step classification model based on backward regression general linear modeling (BRGLM). Table 1 summarizes the reported studies about NAC response prediction for TNBC and other types of breast cancer.

It is noteworthy that most of the reported studies are based on a combination of feature selection and relatively simple models such as Multivariate Logistic Regression (MLR) or Diagonalized Linear Discriminant Analysis (DLDA). However, because there is no clear correlation between the expression of a gene and a NAC response vector (mean PCC =−0.038±0.103), linear models may not be the appropriate choice for classifying the NAC response [28]. In addition, some studies have utilized prior marker information to restrict the search space of optimal markersets, but these studies only utilized the associated genes of predefined mechanisms such as the immune response [29] and the E2F pathway [19,20], which may not fully represent the mechanisms of the NAC response. Moreover, there are only two studies specifically targeting TNBC patients in which pCR prediction by NAC is expected to improve the clinical prognosis.

The Random Forest (RF) is a machine learning model based on an ensemble of decision trees generated by random feature sets [30]. Because it utilizes nonlinear proximity based on bagging of random trees, it can classify samples partitioned by nonlinear decision boundary. It also provides an estimate of the importance of variables in the classification; therefore, if the user wants to reduce the number of variables, it can guide this process according to the importance of the variables. Therefore, it is suitable for developing a gene expression based classification model such as a NAC response prediction model.

To address the aforementioned problems, herein, we propose a novel NAC prediction model based on random forest (RF). We combined prior knowledge of breast cancer disease markers collected from various resources such as the literature, disease databases, drug target databases, and existing marker panels and applied several marker optimization strategies to develop an 86-gene-based RF model that outperforms the reported NAC predictive models in TNBC. To verify data specificity, we performed differential expression analysis and included both prior markers and differentially expressed genes as candidate markers. In addition, the predicted pCR status by the model well explains the distance recurrence free survival (DRFS) of TNBC patients. We also found the associations between the 86 genes and the disease mechanisms of breast cancer such as DNA repair and cell cycle.

There are several issues with reporting a novel NAC response prediction model as follows. First, it is difficult to objectively compare the performance of the existing model and the newly proposed model because there is no definite guideline to select a metric for the performance evaluation. Second, there is no consensus on which model performs best for a given task, because there are no benchmark studies that provide an objective assessment of the performance of NAC response prediction models. Third, it is difficult to replicate the reported studies due to the lack of data and codes used in the reported studies and insufficient reporting on the model development process. Fourth, it was impossible to compare the performance of our model with similarly constructed models in the literature because we could not find any random forest model proposed to predict the NAC response in breast cancer. Therefore, here, we implemented all listed NAC response prediction models in Table 1 and performed a comparative analysis on the same test set.

## 2. Materials and Methods

### 2.1. Collection of Datasets

All gene expression datasets were collected from the GEO database. Table 2 summarizes the number of patients and NAC regimens for each dataset used in this study. As shown in the Table 2, T/FAC (Taxol + Fluorouracil, Anthracycline and cyclophosphamide) and T/FEC (Taxol + Fluorouracil, Epirubicin and cyclophosphamide) were the most used NAC regimens. Here, taxol stands for paclitaxel or docetaxel, and anthracycline stands for doxorubicin or epirubicin, which can be substituted for each other in chemotherapy regimens. The GSE32646 data set contains only patients who received the T/FEC regimen, and all other data sets contain more patients who received the T/FAC regimen. All data included different types of breast cancer, so patients were selected based on receptor status (ER-, PR-, and HER2-). Because GSE25066 had the highest number of TNBC patients, we considered this dataset as the development dataset and the three other datasets as the independent validation datasets (GSE20271, GSE20194, and GSE32646).

### 2.2. Preprocessing

Raw intensity files from the Affymetrix HG U133A or HG U133 Plus 2.0 platforms were processed using the *mas5* function in the *affy* R package [34] to normalize to an average array intensity of 600 and to generate expression values of the probe set level as in Hatzis et al. [18]. Some gene transcripts are recognized as more than one probe set because probe sets represent a set of (perfect match and mismatch) pairs of probes for multiple regions of a single gene transcript sequence. To avoid confusion, we used the average intensity of each probe set that maps to the same gene.

### 2.3. Collection of Prior Breast Cancer Markers

The statistical significance of certain markers in development data sets may not be reproduced due to their low association with breast cancer. It has been reported that prior knowledge approaches can improve the robustness and true biological relevance of selected markers in gene expression datasets [35]. Therefore, in this study, we not only utilized data specific markers such as differentially expressed genes but also included markers associated with breast cancer as candidate markers. To acquire such a prior knowledge markers, we collected breast cancer marker information from the existing literature [36,37,38,39,40,41,42], databases [43,44,45,46], and gene expression marker panels [23,27,47,48,49,50,51,52,53]. We first gathered information on disease genes primarily listed in existing breast cancer reviews through PubMed and google searches. We selected seven papers that provide comprehensive summaries of disease genes associated with breast cancer, as shown in Table 3. Second, we considered the disease gene database DisGeNet [43] and three drug databases (Drug Central [44], Drug Bank [45] and DGIDB [46]) to collect genes and drug targets associated with breast cancer. We also considered genes in existing breast cancer prognostic/responsive marker panels. We considered genes in nine gene expression marker panels including Oncotype Dx [47], Mammaprint [48], Prosigna [27], Endopredict [49], Breast Cancer Index [50], CureBest [51], GenesWellBCT [52], Genomic Grade Index (GGI) [53], and DLDA30 [23]. The gene symbols collected from all resources are standardized by *HGNC Multi Symbol checker* (https://www.genenames.org/tools/multi-symbol-checker/, accessed on 15 November 2021) and mapped to *NCBI Gene IDs* (https://www.ncbi.nlm.nih.gov/gene, accessed on 15 November 2021). In summary, we collected 1079 breast cancer marker genes through this process.

### 2.4. Differential Expression Analysis

We performed a differential expression analysis using the development dataset (GSE25066) to find the most discriminating genes between pCR vs. RD patients. To avoid false positives due to the relatively small sample number and the skewness of individual gene expression levels in the training dataset, we performed bootstrap *t*-test using *boot.t.test* function in the *MKinfer* R package [54]. We utilized bootstrapped *t*-test under unequal variance proposed by Janssen et al. [55], and the number of bootstrap replicates was 9999. We adjusted the *p*-value with the Benjamini-Hotchoberg (BH) method [56] and selected genes with a FDR <0.05 as differentially expressed genes. To avoid randomness of the bootstrap procedure, we iterated the process 10 times and selected genes that showed FDR <0.05 in at least one trial.

### 2.5. Model Training

Because the number of markers is of practical importance for developing cost effective marker panels, all developed marker panels have a relatively small number of genes compared to the number of genes available in the microarray dataset. Therefore, as discussed earlier, we limited the entire search space to the union of prior maker genes (total 1079) and differentially expressed genes (total 52). For Recursive Feature Elimination (RFE), an N of 1000 times was used to derive an optimal set of genes for the RF model (See Supplementary Figure S1). We used 70% of the development dataset as the training set to optimize the marker set. For each trial, we partitioned the training dataset into 3 equal sized folds and trained the RF classifier using 2 folds of the data along with all genes derived from the union of the prior marker genes and the differentially expressed genes. We evaluated the performance of the trained classifier on the remaining fold and evaluated the significance of the feature by the mean decrease of accuracy. After three training and test runs, we derived feature ranks for all genes based on the variable importance and removed the least significant genes from the list of genes. We performed this procedure from the maximum number of genes (1124) to 2 genes and reported the best performing subset. To reduce the search space, here, we searched for the best subset with 10 equally spaced grids, which iteratively search for the best subset when the number of genes in the optimal subset exceeds 100. That is, the trial ends when the number of genes in the optimal subset is less than 100. We repeated this process (N = 1000 times) to obtain candidate models. All models were evaluated on the rest of the test set in the development dataset to find the best performing marker set and RF model. However, because the best model on the Affymetrix U133A dataset did not perform well on the Affymetrix U133 PLUS 2.0 dataset (GSE32646), we considered 30% of the GSE32646 data set as an additional test set to find a robust model for both platforms. To minimize the effect of imbalance in the pCR vs. RD samples, we used Synthetic Minority Oversampling TEchnique (SMOTE) [57] based on the *SMOTE* function in the *DMwR* R package [58]. The *accuracy* was used as a metric to optimize the RF model. In this process, the markers showing the highest accuracy for the test dataset were selected as the marker set for the final model. We used the *rfe* function in the *Caret* [59] R package.

### 2.6. Selection of an Optimal Threshold

Due to the unequal misclassification cost of the pCR prediction, a naive threshold that applies equal probabilities to both pCR and RD may not be an appropriate choice for a pCR prediction model [60]. Because of the unequal misclassification cost, it is recommended to control the type I error (FPR) below a certain level (α) [61]. Here, positive means the desired outcome, i.e., pCR. In other words, when misclassifying RD patients as pCR patients, the cost of the misclassification is higher than vice versa because alternative treatment options for RD patients may be lost. On the other hand, even if pCR is misclassified as RD, the risk of misclassification can be minimized by subsequently reassessing the patient for appropriate treatment options. We therefore selected an optimal threshold based on a predefined false positive rate, α, of ≤10%, which is comparable to an ultrasound (US) image-based pCR prediction [62].

### 2.7. Model Validation

As previously discussed, we used three independent test data sets to validate the proposed RF model. Various binary classification performance metrics were used to evaluate the test performance of the proposed model including Accuracy (ACC), Balanced Accuracy (BACC), Sensitivity (TPR), Specificity (TNR), Precision (PPV), Negative Predictive Value (NPV), F1 score, Metthew Correlation Coefficient (MCC), and Yoden’s Index. Because we predefined α≤10%, the specificity or TNR of the model was about 90%. We also evaluated the AUROC and AUPRC of the proposed model for each test data set and visualized the ROC and PR curves.

### 2.8. Comparative Analysis

We performed comparative analysis of the proposed model with existing NAC response prediction models. We considered ROR-S [27], GGI [53], DLDA30 [23], Hatzis [18], BA100 [26], three-gene [19], five-gene [20], and Immune associated signatures [21] as comparable models for the NAC response prediction, as discussed earlier. To derive the ROR-S and GGI score, we used the *ggi* and *rorS* function in the *genefu* R package [63]. For the DLDA30 score, we reimplemented the DLDA30 score function available at the author’s web site (http://bioinformatics.mdanderson.org/pubdata.html, accessed on 15 November 2021). For the Hatzis model, we used adaptive LASSO based on the genes of the NAC response for ER-breast cancer patients proposed by the authors [18] because model coefficients and a training algorithm (TGDR) were not available. We newly implemented the BA100 [26], three-gene [19], five-gene [20], and immune associated signature [21] models as described in the original papers. Because the BA100 uses a two step classifier, we evaluated the performance of both classifiers separately. We compared the ROC and PR curves, confusion matrices with prespecified FPR and performance metrics of the binary classification.

### 2.9. Survival Analysis

A survival analysis was performed to determine if the proposed model could also predict Distant Recurrence Free Survival (DRFS) in patients receiving NAC. Since only one dataset (GSE25066) provides survival information, we investigated whether the predicted pCR and RD classes had differential survival curves in the training and test datasets. We used the *survfit* function from the *survminer* R package [64] to fit the survival curves of the pCR versus RD on the training and test sets and visualized the survival curves with the *ggsurvplot* function in the same package. The statistical significance of the survival difference was calculated by log-rank test.

### 2.10. Chemosensitivity Analysis

Some projects, such as GDSC [65], CCLE [66], CTRP [67], and PRISM [68] provide TNBC cell line sensitivity to drugs used for T/FAC- or T/FEC-based NAC, i.e., Paclitaxel, Docetaxel, 5-Fluorouracil, Doxorubicin, Epirubicin, and Cyclophosphamide [9]. Because the GDSC project provides the most complete set of sensitivity observations for TNBC cell lines, GDSC profiled cell line sensitivity (AUC of the dose response curves) data were used to investigate the relationship between the pCR probability of the proposed model and the chemosensitivity of TNBC cell lines. To obtain a list of TNBC cell lines, we referred to the study of Chavez et al [69]. Here, we investigated the Spearman rank correlation between the pCR probability and the sensitivity of the TNBC cell lines to these drugs (1 − Area Under the dose response curve, 1−AUC). CCLE microarray data were preprocessed in the same manner as described in Section 2.2 to obtain the pCR scores of the TNBC cell lines. Among 27 TNBC cell lines listed by [69], 16 cell lines are available in the CCLE dataset (BT20, BT549, Cal51, HCC38, HCC1143, HCC1187, HCC1395, HCC1599, HCC1806, HCC1937, HCC2157, Hs578T, MDA-MB-157, MDA-MB-231, MDA-MB-436, and MDA-MB-468). Some drug responses for a particular cell line could be missing; thus, the Spearman’s correlation was calculated by a different number of data points for each drug. For example, only 10 cell lines have a cyclophosphamide drug response, so we calculated the correlation between the pCR probability and drug response from these 10 TNBC cell lines.

### 2.11. Function Enrichment Analysis

To investigate the association between the predictors of our model and cellular function, we performed a functional enrichment analysis [70] based on the Fisher exact test in our in-house module database. Briefly, we collected known gene sets from Gene Ontology [71], MSigDB [72], and Enrichr [73] databases. After removing ambiguous and redundant gene collections from these databases, we built a gene module database of 86,769 unique sets of genes with 29,107 unique genes. An ambiguous gene set here refers to a set of genes that have less direct significance in cellular functions, such as chromosomal location, genome browser PWM, NIH grant related gene sets, etc. [73].

### 2.12. Visualize Error Matrix and Score Distributions

To understand the performance improvement in the proposed 86-gene-based RF model compared to the 8 published NAC response prediction models, we performed several analyses as follows. First, we visualized the sample error matrix according to the prediction of the published models and the proposed model. Second, we visualized the score distribution of the published models and the proposed model to see whether the proposed model has better calibration. Because the score range of the published models differ from each other, we performed min-max normalization as follows:

$$Score_{Norm,i}=\frac{Score_{i}-min\left(Score_{i}\right)}{max\left(Score_{i}\right)-min\left(Score_{i}\right)}$$

The discriminative power of each model was assessed by *t*-test.

## 3. Results

### 3.1. Patient Characteristics

Table 4 shows the characteristics of the patients with TNBC in the development and independent validation datasets. There were no statistically significant differences between the pCR and RD patients for all datasets. Two datasets (GSE20271 and GSE20194) were missing the tumor stage information, and one dataset (GSE32646) was missing the N stage. Stages T2, N0, and Grade 3 had the highest prevalence in the TNBC population in all datasets.

### 3.2. Differential Expression Analysis

**Figure 1:**
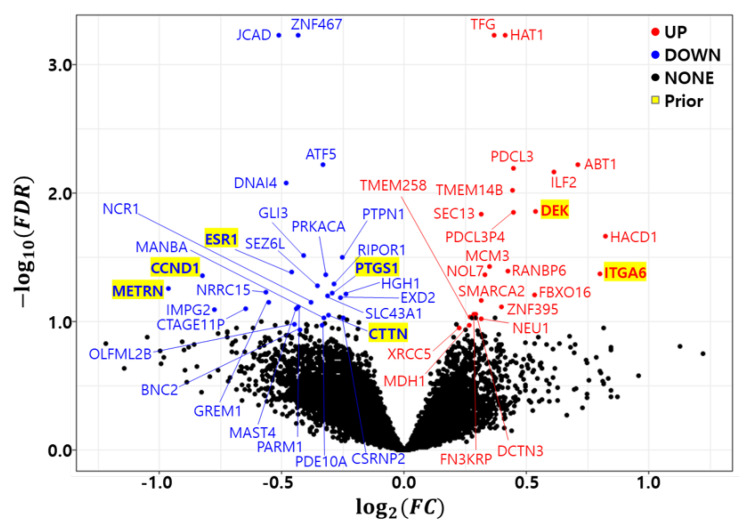
Volcano plot of differentially expressed genes in development dataset (GSE25066). Red: Up regulated genes, Blue: Down regulated genes, Yellow background: Prior marker genes.

As previously discussed, the bootstrap *t*-test was repeated 10 times to minimize the randomness of the bootstrap procedure. A gene with a FDR≤0.05 in at least one trial was called a differentially expressed gene (DEG). Figure 1 shows a volcano plot of the differentially expressed genes (DEGs) in the development dataset (GSE25066). We found 23 up regulated genes (HAT1, TFG, ABT1, PDCL3, ILF2, TMEM14B, DEK, PDCL3P4, SEC13, HACD1, MCM3, RANBP6, ITGA6, NOL7, FBXO16, SMARCA2, ZNF395, FN3KRP, DCTN3, TMEM258, NEU1, MDH1, and XRCC5) and 29 down regulated genes (JCAD, ZNF467, ATF5, DNAI4, GLI3, PTPN1, ESR1, PRKACA, CCND1, RIPOR1, SEZ6L, METRN, LRRC15, PTGS1, HGH1, SLC43A1, EXD2, GREM1, NCR1, PARM1, MAST4, CTAGE11P, IMPG2, CTTN, MANBA, CSRNP2, OLFML2B, PDE10A, and BNC2). Table 5 summarizes the differentially expressed genes (DEGs) in the development dataset. The column *count* indicates how many times a gene was called a DEG in the 10 trials. The Fold Change (FC) and False Discovery Rate (FDR) were averaged from 10 trials and placed in the FC and FDR columns of the table. The number of common genes between the prior marker gene and DEGs was only 7.

### 3.3. Optimal Marker Set and Model Selection by RF-RFE

**Figure 2:**
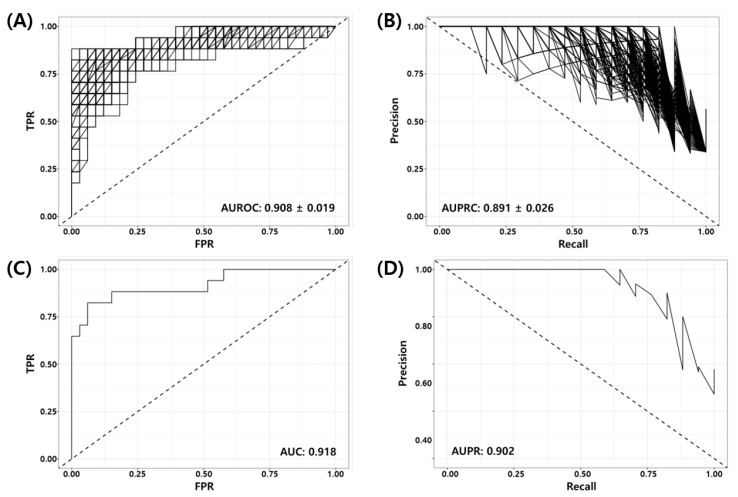
Receiver Operating Characteristic (ROC) and Precision Recall (PR) curves of RF-RFE optimized models for test set (GSE25066, 17 pCR, 33 RD); (**A**) ROC and (**B**) PR curves for all models, (**C**) ROC and (**D**) PR curves for the best model.

Random Forest Recursive Feature Elimination (*RF-RFE*) was used as described in Section 2.5 to derive the optimal combination of markers for the model training from 1124 genes obtained from the union of prior marker genes (1079 genes) and DEGs (52 genes). Here, we used 70% of the development dataset (GSE25066) as the training set (40 pCR vs. 80 RD) and 30% of the data as the test set (17 pCR vs. 33 RD). Figure 2 shows the Receiver Operating Characteristic (ROC) and Precision Recall (PR) curves of the RF-RFE optimized model for the test set. Figure 2A,B show the ROC and PR curves of all 1000 models. It can be seen in the figure that all trained models are working well for the test set (mean BACC = 0.848, AUROC = 0.908±0.019, AUPRC = 0.891±0.026). Figure 2C,D show the ROC and PR curves of the best model consisting of 86 genes (See Supplementary Table S1). The AUROC and AUPRC of the best model were 0.918 and 0.902, respectively. Table 6 shows the binary classification performance metrics for the best model at the predefined threshold of the False Positive Rate (FPR, α≤10%). Here, positive indicates the desired outcome pCR, and negative indicates the undesired outcome RD.

### 3.4. Validation of the Final Model Using Independent Test Datasets

**Figure 3:**
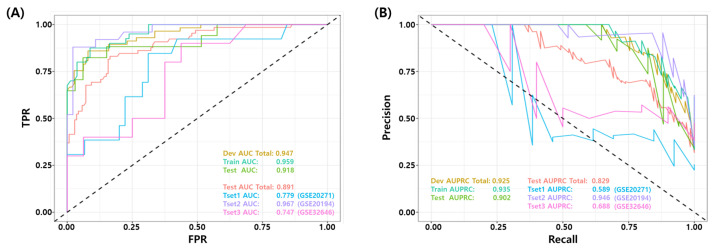
Receiver Operating Characteristic (ROC) and Precision Recall curve of the best models for test set. (GSE20271, GSE20194, GSE32646) (**A**) ROC curve and (**B**) PR curve for independent test datasets.

The final model was validated on three independent data sets (GSE20271, GSE20194, and GSE32646). Figure 3 show the ROC and PR curves of the final model. The test AUROC and AUPRC of the final model for all test samples were 0.891 and 0.829, respectively. The final model performed better on the GSE20194 dataset compared to the other two datasets (GSE20271 and GSE32646), achieving an AUROC 0.967 vs. 0.779 and 0.747, respectively. Table 6 summarizes the binary classification performance of the final model for all test datasets. The average performance for all samples was as follows: BACC 0.800, TPR 0.692, PPV 0.907, NPV 0.864, F1 score 0.732, MCC 0.619, and Yoden’s Index 0.600, respectively. Again, the performance metrics were calculated from a predefined false positive rate (FPR, α≤10%) threshold.

### 3.5. Comparative Analysis

**Figure 4:**
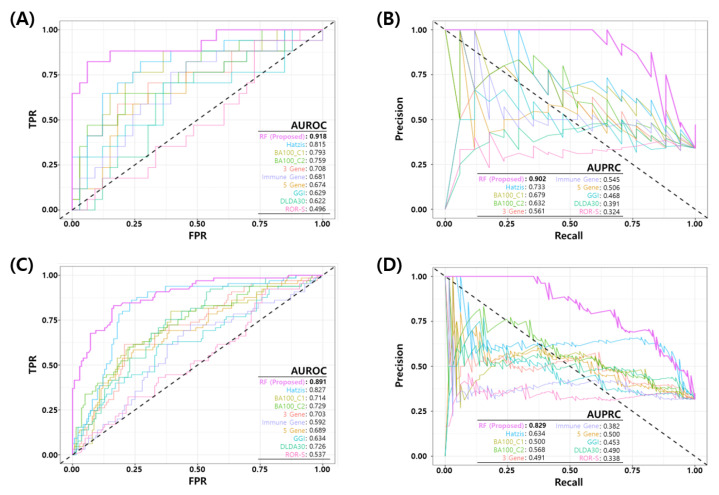
Comparison of Receiver Operating Characteristic (ROC) and Precision Recall (PR) curves of the final model and published NAC prediction models. (**A**) ROC and (**B**) PR curves for the test set in the development dataset (GSE25066). (**C**) ROC and (**D**) PR curves for all test sets in four datasets (development test set + three independent test datasets).

We compared the performance of the proposed RF model with eight published NAC response prediction models, as discussed earlier. Figure 4 shows the ROC and PR curves of proposed RF and all other prediction models. Figure 4A,B show the ROC and PR curves of the test set in the development dataset (GSE25066) and Figure 4C,D show the ROC and PR curves of all the test sets in development and three independent test sets. The proposed model shows a better performance than all the other published models in terms of the AUROC/AUPRC (0.918/0.902 in development test set and 0.891/0.829 in all the test sets). Table 7 shows a comparison of the binary classification performance metrics for the proposed and eight published models at a predefined FPR threshold (α≤10%). For all the test sets, the proposed model shows a superior performance compared to the published models.

### 3.6. Survival Analysis

**Figure 5:**
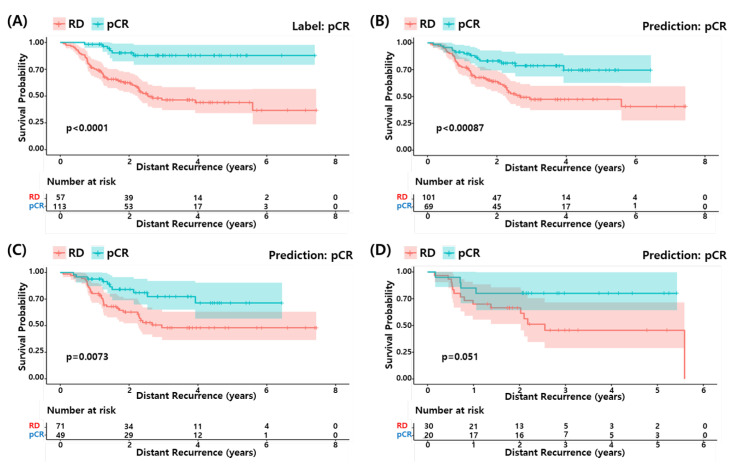
Survival analysis of proposed model. (**A**) pCR label and (**B**) pCR prediction by proposed model in development dataset. (**C**) pCR prediction in the train set and (**D**) pCR prediction in the test set.

We performed survival analysis to determine if the pCR predicted by our model could predict a longer survival of the patients as the actual pCR. Figure 5 shows the survival curves for the actual pCR (A) and predicted pCR in (B) all, (C) training set and (D) test set of the development data (GSE25066). The predicted pCR showed a longer survival compared to the predicted RD patients in all cases. Although the statistical significance of the test set was relatively low due to the small sample size, the pCR predicted by the proposed RF model explains the differences in survival among the TNBC patients well.

### 3.7. Relation to Chemosensitivity in TNBC Cell Models

**Figure 6:**
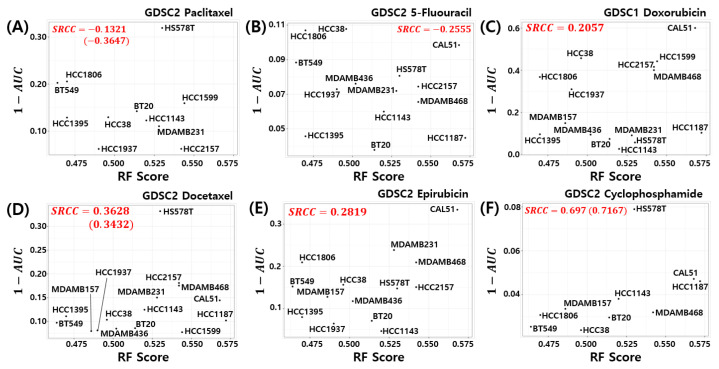
Visualization of pCR scores of the proposed model and chemosensitivity of TNBC cell lines. Six drugs used to treat T/FAC or T/FEC were investigated: (**A**) Paclitaxel, (**B**) 5-FU, (**C**) Doxorubicin, (**D**) Docetaxel, (**E**) Epirubicin, and (**F**) Cyclophosphamide.

**Figure 7:**
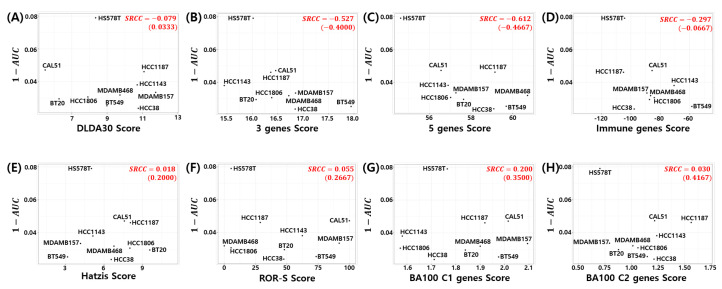
Visualization of pCR score of the seven published models vs. Cyclophosphamide sensitivity in TNBC cell lines: (**A**) DLDA30, (**B**) 3 genes, (**C**) 5 genes, (**D**) Immune genes, (**E**) Hatzis, (**F**) ROR-S, (**G**) BA100 Classifier 1, and (**H**) BA100 Classifier 2. SRCC values in parentheses exclude HS578T data point.

Here, we investigated the relationship between the pCR score of the proposed model and chemosensitivity of the TNBC cell lines. As shown in Figure 6, the pCR score of the proposed model shows the highest Spearman rank correlation for cyclophosphamide sensitivity in the TNBC cell line compared to all other drugs used for the T/FAC or T/FEC treatment (SRCC =0.697, *p*-value =0.031). A high AUC of the dose response curve means a low sensitivity of the cells to the target drug; thus, 1−AUC can be interpreted as the sensitivity score of the drug treated cell line. That is, the proposed model predicts a higher pCR probability when the TNBC cell line has a high sensitivity score to cyclophosphamide. We also investigated the SRCC between the pCR score and the cyclophosphamide chemosensitivity calculated by seven published models; however, as shown in Figure 7, none of the models showed a high positive correlation between the pCR score and the chemosensitivity of the TNBC cell lines. Because the HS578T cell data point appears to be an outlier, we recalculated the SRCC excluding the HS578T data point. As a result, the SRCC increased in all models, but the proposed model (SRCC =0.7167, *p*-value =0.037) showed the highest SRCC. In the case of the BA100 model, the increase in SRCC values was very large when HS578T cells were excluded (SRCC = 0.200, 0.030 vs. 0.3500, 0.4167).

### 3.8. Cellular Functions Associated with the 86 Genes of the Proposed Model

In addition to the performance of the predictive models, a mechanistic understanding of the genes is important for clinical response prediction. To investigate the association between the 86 genes of our model and cellular function, we performed a function enrichment analysis [70] based on Fisher’s exact test on our in house module database.

Table 8 shows the result of the function enrichment analysis. We excluded phenotypic terms, which may cause confusion. Among the terms including five or more genes ($N_{gene}\geq 5$), cases in which 30% or more of the genes of the corresponding term were mapped to the query genes were selected ($Ratio_{mapped}\geq 0.3$). The results show an enrichment of DNA repair and cell cycle gene modules reported to be involved in the NAC response of TNBC [74,75].

### 3.9. Visualization of the Error Matrix and Score Distributions

**Figure 8:**
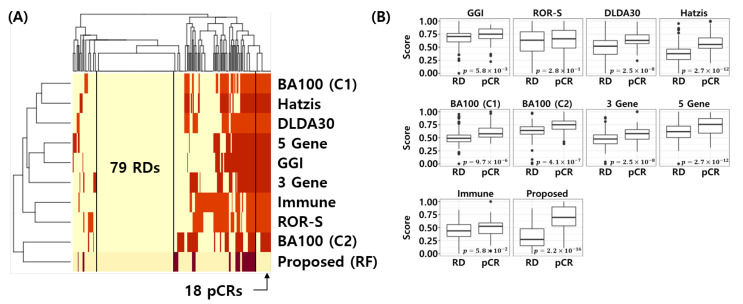
Visualization of error matrix and score distribution of NAC response prediction models. (**A**) Heatmap of the error matrix. Yellow represents correctly classified samples and red represents misclassified samples. (**B**) Score distribution of NAC response prediction models. *t*-test is used to calculate *p*-value for difference between pCR and RD score of NAC response prediction models.

Figure 8A shows the error matrix of the published NAC response prediction models and proposed model. Here, yellow represents correctly classified samples and red represents misclassified samples for each model. Interestingly, all models are correctly classifying 79 RD patients and the proposed model correctly classifies the 18 pCR samples, which were misclassified by all 8 models. Figure 8B shows the min-max normalized score distribution of the NAC response prediction models for all test samples. *t*-test was used to calculate the *p*-value of the score difference between pCR and RD. This analysis reconfirms that the proposed model is most discriminative compared to all other models (*t*-test *p*-value < 2.2 × 10${}^{-16}$) In other words, the proposed model provides a better score distribution, which indicates a better calibration of the model that can separate pCR from RD patients more effectively.

### 3.10. Association for Metabolic Pathway Based Subtypes

**Figure 9:**
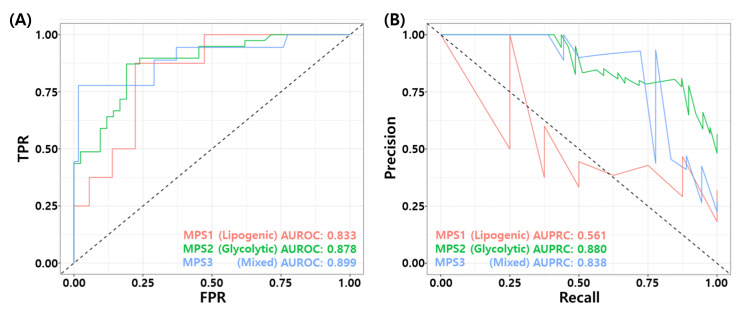
MPS specific performance of the final RF model. (**A**) ROC curves and (**B**) PR curves.

Recently, Gong et al. proposed metabolic-pathway based subtyping (MPS) of triple negative breast cancer to reveal potential therapeutic targets [76]. We stratified our test samples based on the MPS signatures suggested by Gong et al., and evaluate MPS specific performance of the proposed model. We performed GSVA for these MPS genesets on our test datasets. Among 205 samples, 44 samples were classified as MPS1, 81 samples were classified as MPS2, and 80 samples were classified as MPS3. MPS1 and MPS3 samples consist mostly of RD patients. In particular, 81% of MPS1 samples (36/42), and 77.5% of MPS3 samples are RD patients. MPS2 subtype contains 48% of pCR patients (39/81). Figure 9A,B shows the ROC and PR curves for MPS specific prediction by the proposed RF model. The proposed model showed relatively high performance in MPS2 and MPS3 subtypes compared to MPS1 subtype. Table 9 shows the binary classification performance of the proposed model for each MPS subtypes. The BACC of MPS1, MPS2, and MPS3 were 0.646, 0.747, and 0.841, respectively. These results may imply that the TNBC patients who have MPS 2 subtype can have higher probability of pCR with T/FAC or T/FEC based NAC.

## 4. Discussion

In this study, we developed a gene expression based NAC response prediction model using random forest recursive feature elimination (RF-RFE). Extensive characterization of the model performance using ROC, PR curves, and binary classification metrics at a predefined FPR threshold of 10% showed that the proposed 86 gene-based RF model outperforms the existing 8 NAC response predictive models. In addition, we investigated the relationship between the proposed model and drug sensitivity in the TNBC cell models. Our model shows a high Spearman’s rank correlation with cyclophosphamide sensitivity in TNBC cell lines. Through function enrichment analysis, we found that genes in our model are associated with DNA repair and cell cycle mechanisms.

Compared to prognostic models for ER+/HER2- patients currently used in clinical practice to evaluate the benefits of additional adjuvant chemotherapy [77], neoadjuvant chemotherapy response prediction models for TNBC patients are less convincing for use in clinical practice. The major concerns for these predictive models for NAC response is the misclassification of RD patients as pCR. This is because it not only guides overtreatment but also eliminates alternative treatment options for those patients. In other words, there is an unequal classification risk between the type I and type II errors, and in order to objectively evaluate the NAC response prediction models, it is necessary to control the type I error (FPR) below a certain level. We set this level as less than or equal to 10% and set the threshold based on the test set in the development dataset (GSE25066). The level selected in this study is similar to other types of NAC response prediction models, such as clinical variable based models [78], Ultrasound [79,80], and MRI imaging based [81] NAC response prediction models.

We also performed a literature survey for the association between NAC response and predictors of our model, in terms of their enriched mechanisms. Specifically, the genes mapped to the DNA repair term were MSH2 and MSH6. These genes have been reported to be associated with the NAC response in breast cancer [82,83,84]. Knockdown of MYCN has been reported to be associated with the NAC response of TNBC. [85]. Loss of ENO1 has been reported to correlate with clone forming unit (CFU) potential in MDA-MB-231 and BT-549 cell models associated with susceptibility to paclitaxel [86]. RAD51 [87] has been reported to be associated with homologous recombination deficiency (HRD) of TNBC, which affects the NAC response. Inhibition of CDK2 has been reported to be associated with the chemosensitivity of TNBC cell lines [88]. Inhibition of CDK2 has also been reported to be associated with growth inhibition in TNBC bearing mice [89] and reduced cell migration in TNBC cell lines [90]. E2F3 has been reported to be associated with doxorubicin responses [91] and EMT mechanisms in TNBC [92]. MCM2 and MCM3 have also been reported to have a role in DNA repair mechanisms in breast cancer [93], and these genes have also been reported as predictors of other NAC response predictive models [19,20]. The survey may indicate a possible mechanism of the NAC response in TNBC as DNA repair and cell cycle mechanisms by investigating the relationship between genes in the proposed model and the NAC response mechanism in TNBC.

Recently, Gong et al. proposed stratification of the TNBC patients using metabolic pathway enrichment based subtpying (MPS) [76]. They proposed three distinct subtypes, so called MPS1 (Lipogenic), MPS2 (Glycolytic) and MPS3 (Mixed) and validated their results through xenograft and organoid experiments. To derive the subtype, they performed geneset variation analysis (GSVA) for 86 metabolic pathway gene sets and consensus clustering on the GSVA score matrix. The proposed model best explained MPS2 model, which contains a large number of pCR patients compared to other two MPS subtypes. Because the proposed model is associated with DNA Repair mechanisms, the high performance of the proposed model in MPS2 subtype characterized by upregulation of carbohydrate and nucleotide metabolic pathways can be considered as consistent results with the report of Gong et al.

Although this study shows the utility of the proposed RF model, there are some limitations. First, we only utilized Affymetrix microarray datasets, so the proposed model may not be applicable to other platforms such as other microarray platforms and RNA sequencing datasets. Second, due to the limited number of available samples, the performance evaluated by the current study may be inconclusive. However, our results showed fairly consistent results across multiple cohorts. Third, the relationship between the chemosensitivity of TNBC cell lines and pCR probability drawn by the proposed model was only validated by some of the TNBC cell lines available in the GDSC datasets. Because drug sensitivity profiling projects are continuously expanding, we expect validation of the current results with updated drug profiling of the TNBC cell lines in near future.

## 5. Conclusions

In conclusion, the proposed 86 gene-based random forest model provides an accurate prediction of pCR after NAC in triple negative breast cancer and may be associated with DNA repair and cell cycle mechanisms. Our study suggests that the random forest based prediction model can provide a reliable prediction of the clinical response to neoadjuvant chemotherapy and may explain chemosensitivity in TNBC.

## Figures and Tables

**Table 1:** Summary of NAC response prediction studies.

Study	Abb.	$N_{\mathrm{gene}}$	Target Population	Preprocessing	Batch Correction	Performance (AUC)
Hess et al., 2006, JCO [23]	DLDA30	30	All	dChip	Not Reported	0.877
Parker et al., 2009, JCO [27]	ROR-S	52	All	Not Reported	Not Reported	0.781
Liedtke et al., 2009, JCO [22]	GGI	97	All	Not Reported	Not Reported	0.735
Hatzis et al., 2011, JAMA [18]	Hatzis	206	HER2-	MAS5	Not Reported	Not Reported
Fournier et al., 2019, Sci. Rep. [26]	BA100	32	TNBC	MAS5	ComBat, QtNorm	Not Reported
Masanori et al., 2021, Cancers [19]	3 gene	3	TNBC	Not Reported	Not Reported	0.735
Masanori et al., 2021, Am. J. Cancer Res. [20]	5 gene	5	ER+/HER2-	Not Reported	Not Reported	0.813
Changfang Fu et al., 2021, Front. Immunol. [21]	Immune gene	25	All	RMA	ComBat	0.956

**Table 2:** Summary of gene expression datasets used in this study.

Study	GSE	GPL	Platform	NAC Regimen	pCR	RD	Total
Hatzis et al., 2011, JAMA [18]	GSE25066	GPL96	Affymetrix HG U133A	T/FAC (75%), T/FEC (18%), other (7%)	57	113	170
Tabchy A et al., 2010, Clin. Cancer. Res. [31]	GSE20271	GPL96	Affymetrix HG U133A	T/FAC (36.2%), T/FEC (27.6%), FAC (10.3%), FEC (17.2%), other (1.70%)	13	45	58
Shi L et al., 2010, Nat. Biotech. [32]	GSE20194	GPL96	Affymetrix HG U133A	T/FAC (67.6%), T/FEC (16.9%), FAC (4.23%), FEC (1.41%), other (9.87%)	25	46	71
Miyake T et al., 2012, Cancer Sci. [33]	GSE32646	GPL570	Affymetrix HG U133 Plus 2.0	T/FEC (100%)	10	16	26

**Table 3:** Summary of existing publications providing breast cancer marker genes.

Year	Authors	Title	Journal	References
2019	Nadia Harbeck et al.	Breast Cancer	Nat. Rev. Dis. Primers	PMID: 31548545 [36]
2019	Francois Bertucci et al.	Genomic Characterization of Metastatic Breast Cancers	Nature	PMID: 31118521 [37]
2018	Francisco Sanchez-Vega et al.	Oncogenic Signaling Pathways in The Cancer Genome Atlas	Cell	PMID: 29625050 [38]
2018	Chandra P. Leo et al.	Breast Cancer Drug Approvals by the US FDA From 1949 to 2018	NRDD	PMID: 31907423 [39]
2016	Xiaofeng Dai et al.	Cancer Hallmarks, Biomarkers and Breast Cancer Molecular Subtypes	J. Cancer	PMID: 27390604 [40]
2016	Serena Nik-Zainal et al.	Landscape of Somatic Mutations in 560 Breast Cancer Whole-Genome Sequences	Nature	PMID: 27135926 [41]
2012	Cancer Genome Atlas Network	Comprehensive Molecular Portraits of Human Breast Tumours	Nature	PMID: 23000897 [42]

**Table 4:** Patient characteristics of the development (1 Dataset—GSE25066) and Validation Datasets (3 Datasets—GSE20271, GSE20194, GSE32646).

Characteristic	GSE25066			GSE20271			GSE20194			GSE32646		
	**pCR**	**RD**	***p*-Value**	**pCR**	**RD**	***p*-Value**	**pCR**	**RD**	***p*-Value**	**pCR**	**RD**	***p*-Value**
N	57	113		13	45		25	46		10	16	
Age (Med, IQR)	48 (41–53)	50 (40–60)	0.1	53 (49–58)	51 (40–58)	0.47	48 (44–53)	51 (42–61)	0.17	60 (54–67)	56 (43–63)	0.46
T Stage			0.28			0.42			0.95			0.73
T1	3 (5%)	5 (4%)		0 (0%)	1 (2%)		2 (8%)	4 (9%)		1 (10%)	0 (0%)	
T2	32 (56%)	47 (42%)		8 (62%)	15 (33%)		13 (52%)	20 (43%)		8 (80%)	12 (75%)	
T3	15 (26%)	38 (34%)		2 (15%)	13 (29%)		5 (20%)	9 (20%)		1 (10%)	3 (19%)	
T4	7 (12%)	23 (20%)		3 (23%)	16 (36%)		5 (20%)	12 (26%)		0 (0%)	1 (6%)	
Missing	0 (0%)	0 (0%)		0 (0%)	0 (0%)		0 (0%)	1 (2%)				
N Stage			0.86			0.61			0.32			NA
N0	15 (26%)	26 (23%)		4 (31%)	14 (31%)		2 (8%)	9 (20%)				
N1	26 (46%)	52 (46%)		7 (54%)	16 (36%)		16 (64%)	18 (39%)				
N2	8 (14%)	21 (19%)		2 (15%)	12 (27%)		3 (12%)	8 (17%)				
N3	8 (14%)	14 (12%)		0 (0%)	3 (7%)		3 (12%)	7 (15%)				
Missing	0 (0%)	0 (0%)		0 (0%)	0 (0%)		1 (4%)	4 (9%)				
Grade			0.34			1			0.19			0.32
G1	0 (0%)	1 (1%)		0 (0%)	1 (2%)		0 (0%)	0 (0%)		0 (0%)	1 (6%)	
G2	4 (7%)	16 (14%)		2 (15%)	6 (13%)		2 (8%)	9 (20%)		4 (40%)	10 (62%)	
G3	48 (84%)	86 (76%)		9 (69%)	26 (58%)		22 (88%)	31 (67%)		6 (60%)	5 (31%)	
Missing	5 (9%)	10 (9%)		2 (15%)	12 (27%)		1 (4%)	6 (13%)		0 (0%)	0 (0%)	
Tumor Stage			0.79			NA			NA			0.54
I	1 (2%)	2 (2%)								0 (0%)	0 (0%)	
IIA	10 (18%)	15 (13%)								1 (10%)	4 (25%)	
IIB	20 (35%)	34 (30%)								8 (80%)	8 (50%)	
IIIA	11 (19%)	32 (28%)								1 (10%)	3 (19%)	
IIIB	10 (18%)	21 (19%)								0 (0%)	1 (6%)	
IIIC	5 (9%)	7 (6%)								0 (0%)	0 (0%)	
Inflammatory	0 (0%)	2 (2%)								0 (0%)	0 (0%)	

pCR: pathlogical Complete Response, RD: Residual Disease.

**Table 5:** Differentially expressed prior marker genes between pCR vs. RD in development dataset (GSE25066). Bold text indicates prior marker genes.

Symbol	NCBI ID	Count	FC	FDR	Direction	Symbol	NCBI ID	Count	FC	FDR	Direction
HAT1	8520	10	1.330	0.00	Up	LRRC15	131578	3	0.676	5.89 × 10${}^{-2}$	Down
TFG	10342	10	1.288	0.00	Up	PTGS1	5742	3	0.816	6.03 × 10${}^{-2}$	Down
JCAD	57608	10	0.701	0.00	Down	HGH1	51236	3	0.847	6.05 × 10${}^{-2}$	Down
ZNF467	168544	10	0.740	0.00	Down	FBXO16	157574	3	1.447	6.16 × 10${}^{-2}$	Up
ATF5	22809	9	0.795	5.97 × 10${}^{-3}$	Down	SLC43A1	8501	3	0.806	6.25 × 10${}^{-2}$	Down
ABT1	29777	9	1.636	5.97 × 10${}^{-3}$	Up	EXD2	55218	2	0.834	6.47 × 10${}^{-2}$	Down
PDCL3	79031	9	1.361	6.39 × 10${}^{-3}$	Up	SMARCA2	6595	2	1.243	6.80 × 10${}^{-2}$	Up
ILF2	3608	9	1.528	6.87 × 10${}^{-3}$	Up	GREM1	26585	2	0.681	7.02 × 10${}^{-2}$	Down
DNAI4	79819	9	0.716	8.33 × 10${}^{-3}$	Down	NCR1	9437	2	0.768	7.05 × 10${}^{-2}$	Down
TMEM14B	81853	9	1.360	9.48 × 10${}^{-3}$	Up	PARM1	25849	2	0.741	7.67 × 10${}^{-2}$	Down
DEK	7913	8	1.450	1.38 × 10${}^{-2}$	Up	ZNF395	55893	2	1.317	7.72 × 10${}^{-2}$	Up
PDCL3P4	285359	8	1.361	1.41 × 10${}^{-2}$	Up	MAST4	375449	1	0.737	7.90 × 10${}^{-2}$	Down
SEC13	6396	8	1.244	1.45 × 10${}^{-2}$	Up	CTAGE11P	647288	1	0.638	7.94 × 10${}^{-2}$	Down
HACD1	9200	7	1.768	2.15 × 10${}^{-2}$	Up	IMPG2	50939	1	0.585	8.02 × 10${}^{-2}$	Down
GLI3	2737	6	0.752	3.03 × 10${}^{-2}$	Down	FN3KRP	79672	1	1.218	8.72 × 10${}^{-2}$	Up
PTPN1	5770	6	0.838	3.15 × 10${}^{-2}$	Down	DCTN3	11258	1	1.225	8.80 × 10${}^{-2}$	Up
MCM3	4172	6	1.274	3.71 × 10${}^{-2}$	Up	CTTN	2017	1	0.808	8.93 × 10${}^{-2}$	Down
RANBP6	26953	5	1.341	4.04 × 10${}^{-2}$	Up	TMEM258	746	1	1.205	9.16 × 10${}^{-2}$	Up
ESR1	2099	5	0.728	4.13 × 10${}^{-2}$	Down	MANBA	4126	1	0.798	9.35 × 10${}^{-2}$	Down
ITGA6	3655	5	1.740	4.23 × 10${}^{-2}$	Up	CSRNP2	81566	1	0.842	9.40 × 10${}^{-2}$	Down
NOL7	51406	4	1.256	4.32 × 10${}^{-2}$	Up	NEU1	4758	1	1.245	9.44 × 10${}^{-2}$	Up
PRKACA	5566	4	0.801	4.34 × 10${}^{-2}$	Down	OLFML2B	25903	1	0.733	1.04 × 10${}^{-1}$	Down
CCND1	595	4	0.564	4.41 × 10${}^{-2}$	Down	MDH1	4190	1	1.203	1.06 × 10${}^{-1}$	Up
RIPOR1	79567	4	0.820	5.07 × 10${}^{-2}$	Down	PDE10A	10846	1	0.793	1.06 × 10${}^{-1}$	Down
SEZ6L	23544	4	0.782	5.23 × 10${}^{-2}$	Down	XRCC5	7520	1	1.167	1.12 × 10${}^{-1}$	Up
METRN	79006	3	0.513	5.56 × 10${}^{-2}$	Down	BNC2	54796	1	0.744	1.15 × 10${}^{-1}$	Down

**Table 6:** Binary classification performance of the final model for all test sets.

Dataset	pCR (Positive)	RD (Negative)	TP	FP	FN	TN	ACC	BACC	TPR	TNR	PPV	NPV	FNR	F1	MCC	Yoden’s Index	AUROC	AUPRC
GSE25066	17	33	14	3	3	30	0.880	0.866	0.824	0.909	0.909	0.909	0.177	0.824	0.733	0.733	0.918	0.902
GSE20271	13	45	5	4	8	41	0.793	0.648	0.385	0.911	0.911	0.837	0.615	0.455	0.341	0.296	0.779	0.589
GSE20194	25	46	22	4	3	42	0.901	0.897	0.880	0.913	0.913	0.933	0.120	0.863	0.786	0.793	0.967	0.946
GSE32646	10	16	4	1	6	15	0.731	0.669	0.400	0.938	0.938	0.714	0.600	0.533	0.417	0.338	0.747	0.688
Total	65	140	45	13	20	127	0.839	0.800	0.692	0.907	0.907	0.864	0.308	0.732	0.619	0.599	0.891	0.829

TP: True Positive, FP: False Positive, FN: False Negative, TN: True Negative, ACC: Accuracy, BACC: Balanced Accuracy, TPR: True Positive Rate, TNR: True Negative Rate, PPV: Positive Predictive Value, NPV: Negative Predictive Value, FNR: False Negative Rate, MCC: Mathew Correlation Coefficient, AUROC: Area Under Reciever Operating Characteristic Curve, AUPRC: Area Under Precision Recall Curve. Here, positive samples refer to the pCR samples.

**Table 7:** Comparison of binary classification performance of best model and published NAC prediction models.

Dataset	pCR	RD	TP	FP	FN	TN	ACC	BACC	TPR	TNR	PPV	NPV	FNR	F1	MCC	Yoden’s Index	AUROC	AUPRC	Method
Test Set (GSE25066)	17	33	5	2	12	31	0.720	0.617	0.294	0.939	0.939	0.721	0.706	0.417	0.319	0.234	0.629	0.468	GGI
			2	3	15	30	0.640	0.513	0.118	0.909	0.909	0.667	0.882	0.182	0.042	0.027	0.496	0.324	ROR-S
			7	2	10	31	0.760	0.676	0.412	0.939	0.939	0.756	0.588	0.539	0.433	0.351	0.815	0.733	Hatzis
			7	2	10	31	0.760	0.676	0.412	0.939	0.939	0.756	0.588	0.539	0.433	0.351	0.708	0.561	3 Gene
			2	1	15	32	0.680	0.544	0.118	0.970	0.970	0.681	0.882	0.200	0.174	0.087	0.674	0.506	5 Gene
			4	3	13	30	0.680	0.572	0.235	0.909	0.909	0.698	0.765	0.333	0.197	0.144	0.681	0.545	Immune
			7	2	10	31	0.760	0.676	0.412	0.939	0.939	0.756	0.588	0.539	0.433	0.351	0.793	0.679	BA100 C1
			8	2	9	31	0.780	0.705	0.471	0.939	0.939	0.775	0.529	0.593	0.486	0.410	0.759	0.632	BA100 C2
			1	3	16	30	0.620	0.484	0.059	0.909	0.909	0.652	0.941	0.095	-0.056	-0.032	0.622	0.391	DLDA30
			14	2	3	31	0.900	0.882	0.824	0.939	0.939	0.912	0.177	0.849	0.775	0.763	0.918	0.902	Proposed
All Testsets	65	141	12	14	53	127	0.675	0.543	0.185	0.901	0.901	0.706	0.815	0.264	0.119	0.085	0.606	0.406	GGI
			9	14	56	127	0.660	0.520	0.139	0.901	0.901	0.694	0.862	0.205	0.058	0.039	0.545	0.344	ROR-S
			24	14	41	127	0.733	0.635	0.369	0.901	0.901	0.756	0.631	0.466	0.323	0.270	0.827	0.634	Hatzis
			16	12	49	129	0.704	0.581	0.246	0.915	0.915	0.725	0.754	0.344	0.218	0.161	0.705	0.490	3 Gene
			18	13	47	128	0.709	0.592	0.277	0.908	0.908	0.731	0.723	0.375	0.240	0.185	0.691	0.499	5 Gene
			9	14	56	127	0.660	0.520	0.139	0.901	0.901	0.694	0.862	0.205	0.058	0.039	0.593	0.381	Immune
			19	14	46	127	0.709	0.597	0.292	0.901	0.901	0.734	0.708	0.388	0.245	0.193	0.716	0.500	BA100 C1
			26	14	39	127	0.743	0.650	0.400	0.901	0.901	0.765	0.600	0.495	0.353	0.301	0.729	0.567	BA100 C2
			15	13	50	128	0.694	0.569	0.231	0.908	0.908	0.719	0.769	0.323	0.188	0.139	0.728	0.490	DLDA30
			45	13	20	128	0.840	0.800	0.692	0.908	0.908	0.865	0.308	0.732	0.620	0.600	0.892	0.829	Proposed

TP: True Positive, FP: False Positive, FN: False Negative, TN: True Negative, ACC: Accuracy, BACC: Balanced Accuracy, TPR: True Positive Rate, TNR: True Negative Rate, PPV: Positive Predictive Value, NPV: Negative Predictive Value, FNR: False Negative Rate, MCC: Mathew Correlation Coefficient, AUROC: Area Under Reciever Operating Characteristic Curve, AUPRC: Area Under Precision Recall Curve. Here, positive samples refer to the pCR samples.

**Table 8:** Cellular function associated with 86 genes of the RF model. $N_{gene}$: the number of gene in the term. $N_{mapped}$: the number of mapped query genes to the term. $ratio_{mapped}$ The ratio of gene in the term mapped with query genes, i.e., $N_{mapped}/N_{gene}$.

SRC DB	Geneset Name	Category	$N_{\mathrm{gene}}$	$N_{\mathrm{mapped}}$	${\mathrm{ratio}}_{\mathrm{mapped}}$	*p*-Value	Gene Mapped
Gene Ontology	GO:0043570: maintenance of DNA repeat elements	Cell_Function	5	2	0.400	8.78 × 10${}^{-5}$	*MSH2*, *MSH6*
Gene Ontology	GO:0032135: DNA insertion or deletion binding	Cell_Function	6	2	0.333	1.31 × 10${}^{-4}$	*MSH2*, *MSH6*
Jensen_COMPARTMENTS	Mismatch_repair_complex	Cell_Localization	6	2	0.333	1.31 × 10${}^{-4}$	*MSH2*, *MSH6*
TF_Perturbations_Followed_by_Expression	MYCN_SHRNA_IMR575_HUMAN_GSE80397_6HR_RNASEQ_UP	Transcription Factor	6	2	0.333	1.31 × 10${}^{-4}$	*ENO1*, *MCM3*
CORUM	MSH2/6-BLM-p53-RAD51 complex (human)	Protein_Complex	5	3	0.600	2.57 × 10${}^{-7}$	*RAD51*, *MSH2*, *MSH6*
CORUM	PCNA-MutS-alpha-MutL-alpha-DNA complex (human)	Protein_Complex	5	2	0.400	8.78 × 10${}^{-5}$	*MSH2*, *MSH6*
CORUM	MCM complex (human)	Protein_Complex	6	2	0.333	1.31 × 10${}^{-4}$	*MCM2*, *MCM3*
Reactome	R-HSA-68911: G2 Phase	Pathway	5	2	0.400	8.78 × 10${}^{-5}$	*CDK2*, *E2F3*

SRC DB: Source Database, $N_{gene}$: Number of genes annotated for the geneset, $N_{mapped}$: Number of mapped genes from query genes, $ratio_{mapped}$: $N_{mapped}/N_{gene}$, *p*-value: Fisher’s Exact test *p*-value.

**Table 9:** Comparison of metabolic pathway based subtype (MPS) specific performance of the proposed RF model.

MPS Subtypes	pCR	RD	TP	FP	FN	TN	ACC	BACC	TPR	TNR	PPV	NPV	FNR	F1	MCC	Yoden’s Index	AUROC	AUPRC
MPS1 (Lipogenic)	8	36	3	3	5	33	0.818	0.646	0.375	0.917	0.917	0.868	0.625	0.429	0.328	0.292	0.833	0.561
MPS2 (Glycolytic)	39	42	23	4	16	38	0.753	0.747	0.590	0.905	0.905	0.704	0.410	0.697	0.524	0.495	0.878	0.880
MPS3 (Mixed)	18	62	14	6	4	56	0.875	0.841	0.778	0.903	0.903	0.933	0.222	0.737	0.657	0.681	0.899	0.838

TP: True Positive, FP: False Positive, FN: False Negative, TN: True Negative, ACC: Accuracy, BACC: Balanced Accuracy, TPR: True Positive Rate, TNR: True Negative Rate, PPV: Positive Predictive Value, NPV: Negative Predictive Value, FNR: False Negative Rate, MCC: Mathew Correlation Coefficient, AUROC: Area Under Reciever Operating Characteristic Curve, AUPRC: Area Under Precision Recall Curve. Here, positive samples refer to the pCR samples.

## Data Availability

The GEO datasets analyzed this study are available in the Gene Expression Omnibus repository, https://www.ncbi.nlm.nih.gov/geo/query/acc.cgi?acc=GSE25066, https://www.ncbi.nlm.nih.gov/geo/query/acc.cgi?acc=GSE20271, https://www.ncbi.nlm.nih.gov/geo/query/acc.cgi?acc=GSE20194, https://www.ncbi.nlm.nih.gov/geo/query/acc.cgi?acc=GSE32646 (accessed on 15 November 2021).

## Supplementary Materials

The following supporting information can be downloaded at: https://www.mdpi.com/article/10.3390/cancers14040881/s1, Figure S1: Flowchart of model training and testing. Table S1: All 86 predictors of the final RF model and their feature importances.
