# Supplementary material for: Development of Gene Expression-Based Random Forest Model for Predicting Neoadjuvant Chemotherapy Response in Triple-Negative Breast Cancer
Source: Cancers (Basel). 2022 Feb 10;14(4):881. doi: 10.3390/cancers14040881 (PMC8870575; doi:10.3390/cancers14040881)
Supplement: Supplementary file 1 [file cancers-14-00881-s001.zip › cancers-1516893-supplementary.pdf]

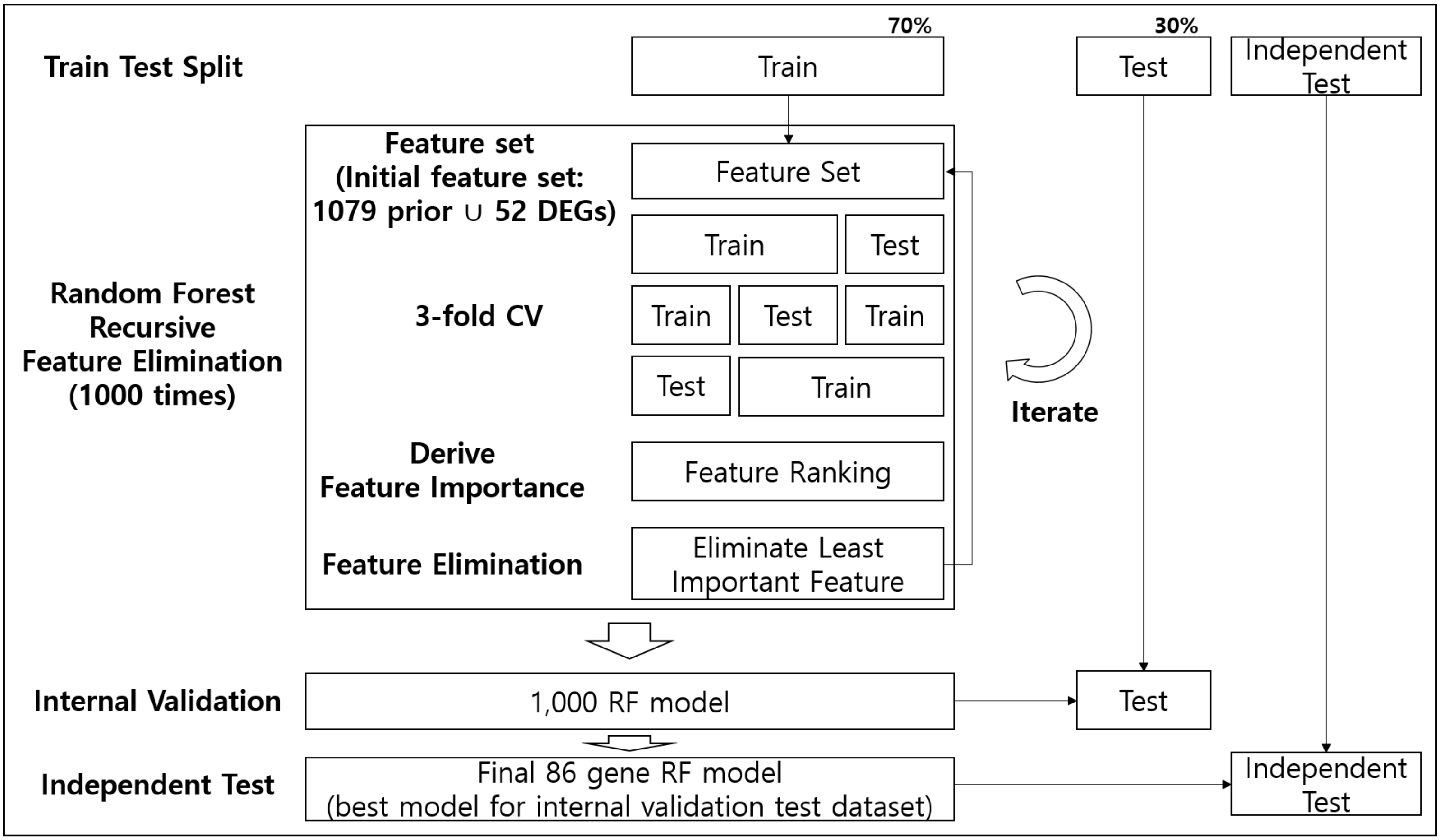

Figure S1. Model training and testing. We performed random forest recursive feature elimination from 1,000 random subsets of features using 70% of the developmental dataset. The initial feature set is union of 1,079 prior marker genes and 52 differentially expressed genes. RF-RFE removes the least important feature for each iteration from the various 3-fold cross validation datasets and optimizes features until less than 100 features remain. After training of 1,000 RF models, we tested trained models on remaining 30% of the developmental dataset and selected best RF model based on the model performance on the test set. Finally, we assessed performance of the final RF model on the independent test dataset to estimate applicability of our model on unseen dataset.

N	Entrez	Symbol	NAME	MDA_in_RD	MDA_in_pCR	MDA_Avg	MDG
1	10342	TFG	Trafficking From ER To Golgi Regulator	0.0231	0.0301	0.0263	4.7242
2	1031	CDKN2C	Cyclin Dependent Kinase Inhibitor 2C	0.0078	0.0109	0.0093	1.9133
3	22809	ATF5	Activating Transcription Factor 5	0.0114	0.0109	0.011	2.2093
4	92140	MTDH	Metadherin	0.005	0.0051	0.005	0.9313
5	26953	RANBP6	Ran Binding Protein 6	0.0098	0.0173	0.0133	2.0716
6	9200	HACD1	3-Hydroxyacyl-Coa Dehydratase 1	0.0071	0.0081	0.0075	0.9431
7	1374	CPT1A	Carnitine Palmitoyltransferase 1A	0.008	0.0069	0.0074	1.6286
8	79006	METRN	Meteorin, Glial Cell Differentiation Regulator	0.0091	0.0101	0.0096	1.8404
9	647288	CTAGE11P	Ctage Family Member 11, Pseudogene	0.006	0.0049	0.0054	1.4074
10	6817	SULT1A1	Sulfotransferase Family 1A Member 1	0.0085	0.007	0.0076	1.4552
11	7295	TXN	Thioredoxin	0.0073	0.0054	0.0063	1.1675
12	2877	GPX2	Glutathione Peroxidase 2	0.0094	0.0086	0.0088	1.4503
13	4126	MANBA	Mannosidase Beta	0.0061	0.004	0.0051	1.3862
14	79567	RIPOR1	Rho Family Interacting Cell Polarization Regulator 1	0.0116	0.0097	0.0108	1.9219
15	6615	SNAI1	Snail Family Transcriptional Repressor 1	0.0044	0.0057	0.0049	0.9636
16	7517	XRCC3	X-Ray Repair Cross Complementing 3	0.0069	0.0066	0.0068	1.2223
17	11258	DCTN3	Dynactin Subunit 3	0.0033	0.003	0.0031	0.8117
18	10010	TANK	Traf Family Member Associated Nfkb Activator	0.0081	0.0089	0.0085	1.4219
19	1365	CLDN3	Claudin 3	0.0037	0.0048	0.0043	0.6875
20	8520	HAT1	Histone Acetyltransferase 1	0.0075	0.0052	0.0063	1.123
21	6396	SEC13	Sec13 Homolog, Nuclear Pore And Copii Coat Complex Component	0.0078	0.0069	0.0071	1.421
22	7296	TXNRD1	Thioredoxin Reductase 1	0.0022	0.0049	0.0035	0.7417
23	8501	SLC43A1	Solute Carrier Family 43 Member 1	0.0078	0.0092	0.0083	1.423
24	29777	ABT1	Activator Of Basal Transcription 1	0.0046	0.0038	0.0041	0.7048
25	4240	MFGE8	Milk Fat Globule Egf And Factor V/Viii Domain Containing	0.0068	0.013	0.0097	1.533
26	65996	CENPBD2P	Cenpb Dna-Binding Domains Containing 2, Pseudogene	0.0031	0.0028	0.0029	0.8238
27	56912	IFT46	Intraflagellar Transport 46	0.005	0.0033	0.0041	0.8478
28	7913	DEK	Dek Proto-Oncogene	0.0083	0.0031	0.0056	1.0529
29	10551	AGR2	Anterior Gradient 2, Protein Disulphide Isomerase Family Member	0.0032	0.0033	0.0032	0.7306
30	50939	IMPG2	Interphotoreceptor Matrix Proteoglycan 2	0.0049	0.0044	0.0047	1.0051
31	81853	TMEM14B	Transmembrane Protein 14B	0.008	0.0029	0.0053	1.4628
32	6774	STAT3	Signal Transducer And Activator Of Transcription 3	0.0035	0.0038	0.0035	0.8189
33	79819	DNAI4	Dynein Axonemal Intermediate Chain 4	0.0037	0.0037	0.0037	0.8687
34	847	CAT	Catalase	0.0038	0.0033	0.0035	0.7694
35	1871	E2F3	E2F Transcription Factor 3	0.0067	0.0047	0.0058	1.0203
36	51236	HGH1	Hgh1 Homolog	0.0057	0.0086	0.0071	1.0748
37	3853	KRT6A	Keratin 6A	0.0016	0.0028	0.0021	0.6581
38	23705	CADM1	Cell Adhesion Molecule 1	0.0051	0.0059	0.0055	1.1834
39	2152	F3	Coagulation Factor Iii, Tissue Factor	0.0049	0.0026	0.0037	0.7488
40	7480	WNT10B	Wnt Family Member 10B	0.0061	0.0082	0.0072	1.415
41	4524	MTHFR	Methylenetetrahydrofolate Reductase	0.0029	0.003	0.0029	0.7042
42	6425	SFRP5	Secreted Frizzled Related Protein 5	0.0031	0.0033	0.0032	0.6614
43	3237	HOXD11	Homeobox D11	0.0097	0.0025	0.006	0.8352
44	2023	ENO1	Enolase 1	0.0032	0.0079	0.0055	0.9109
45	2099	ESR1	Estrogen Receptor 1	0.0056	0.0028	0.0041	0.7452
46	10874	NMU	Neuromedin U	0.0015	0.0031	0.0023	0.5148
47	4548	MTR	5-Methyltetrahydrofolate-Homocysteine Methyltransferase	0.0026	0.0022	0.0023	0.605
48	4330	MN1	Mn1 Proto-Oncogene, Transcriptional Regulator	0.0054	0.0037	0.0044	0.7955
49	5566	PRKACA	Protein Kinase Camp-Activated Catalytic Subunit Alpha	0.003	0.0025	0.0028	0.5481
50	4172	MCM3	Minichromosome Maintenance Complex Component 3	0.0048	0.0023	0.0035	0.7966
51	488	ATP2A2	Atpase Sarcoplasmic/Endoplasmic Reticulum Ca2+ Transporting 2	0.0047	0.0022	0.0034	0.7786
52	285359	PDCL3P4	Pdcl3 Pseudogene 4	0.005	0.0039	0.0045	1.0753
53	4085	MAD2L1	Mitotic Arrest Deficient 2 Like 1	0.0035	0.0019	0.0026	0.534
54	636	BICD1	Bicd Cargo Adaptor 1	0.0042	0.0052	0.0047	0.7729
55	1728	NQO1	Nad(P)H Quinone Dehydrogenase 1	0.0035	0.0032	0.0034	0.7957
56	79031	PDCL3	Phosducin Like 3	0.0051	0.0049	0.005	0.796
57	196	AHR	Aryl Hydrocarbon Receptor	0.0046	0.0037	0.0042	0.7156
58	8659	ALDH4A1	Aldehyde Dehydrogenase 4 Family Member A1	0.0016	0.0063	0.0038	0.6367
59	6595	SMARCA2	Swi/Snf Related, Matrix Associated, Actin Dependent Regulator Of Chromatin, Subfamily A, Member 2	0.0029	0.0052	0.004	0.8504
60	746	TMEM258	Transmembrane Protein 258	0.004	0.0045	0.0041	0.8848
61	8518	ELP1	Elongator Acetyltransferase Complex Subunit 1	0.0025	0.0018	0.0021	0.512
62	5888	RAD51	Rad51 Recombinase	0.0021	0.0018	0.002	0.4581
63	51406	NOL7	Nucleolar Protein 7	0.0031	0.0017	0.0024	0.4844
64	4171	MCM2	Minichromosome Maintenance Complex Component 2	0.0037	0.0034	0.0036	0.7249
65	6160	RPL31	Ribosomal Protein L31	0.0021	0.0016	0.0018	0.4753
66	6194	RPS6	Ribosomal Protein S6	0.0036	0.0029	0.0033	0.5774
67	9052	GPRC5A	G Protein-Coupled Receptor Class C Group 5 Member A	0.0031	0.0031	0.0031	0.6695
68	11260	XPOT	Exportin For Trna	0.0024	0.001	0.0017	0.4242
69	11127	KIF3A	Kinesin Family Member 3A	0.0021	0.0039	0.003	0.5805
70	84617	TUBB6	Tubulin Beta 6 Class V	0.0029	0.0013	0.0021	0.5358
71	3688	ITGB1	Integrin Subunit Beta 1	0.0035	0.003	0.0032	0.6971
72	9898	UBAP2L	Ubiquitin Associated Protein 2 Like	0.0027	0.0017	0.0022	0.3864
73	4660	PPP1R12B	Protein Phosphatase 1 Regulatory Subunit 12B	0.0032	0.0022	0.0027	0.4533
74	55165	CEP55	Centrosomal Protein 55	0.003	0.0035	0.0031	0.6357
75	4436	MSH2	Muts Homolog 2	0.0015	0.0021	0.0018	0.3028
76	203068	TUBB	Tubulin Beta Class I	0.0011	0.0013	0.0012	0.407
77	5352	PLOD2	Procollagen-Lysine,2-Oxoglutarate 5-Dioxygenase 2	0.0008	0.0022	0.0015	0.3952
78	1029	CDKN2A	Cyclin Dependent Kinase Inhibitor 2A	0.0041	0.0017	0.0029	0.6068
79	644	BLVRA	Biliverdin Reductase A	0.0031	0.0028	0.0029	0.557
80	1017	CDK2	Cyclin Dependent Kinase 2	0.001	0.0023	0.0016	0.2981
81	7178	TPT1	Tumor Protein, Translationally-Controlled 1	0.0023	0.0014	0.0019	0.6859
82	2956	MSH6	Muts Homolog 6	0.0024	0.0023	0.0024	0.5586
83	6794	STK11	Serine/Threonine Kinase 11	0.0034	0.0022	0.0028	0.4667
84	7153	TOP2A	DNA Topoisomerase II Alpha	0.0018	0.0009	0.0014	0.2882
85	5984	RFC4	Replication Factor C Subunit 4	0.0019	0.0013	0.0016	0.3538
86	55247	NEIL3	Nei Like DNA Glycosylase 3	0.0015	0.0016	0.0016	0.4189

Table S1. All 86 predictors of the final RF model and their feature importance. MDA: Mean Decrease in Accuracy, MDG: Mean Decrease in Gini index.
